# A Step Forward: About the Progresses Made in the Second Edition of the Special Issue “The Multiple Mechanisms Underlying Neuropathic Pain”

**DOI:** 10.3390/ijms24108590

**Published:** 2023-05-11

**Authors:** Sara Marinelli, Roberto Coccurello

**Affiliations:** 1 National Council of Research (CNR), Institute of Biochemistry and Cell Biology, 00015 Monterotondo, Italy; 2 Institute for Complex Systems (ISC), National Council of Research (CNR), 00185 Rome, Italy; 3 European Center for Brain Research/Santa Lucia Foundation IRCCS, 00143 Rome, Italy

The present editorial intends to comment on the contributions published in the second edition of the Special Issue (SI) “The Multiple Mechanisms Underlying Neuropathic Pain”. Compared to the first edition, some themes, such as the discussion of the role of the Insular cortex (ICx) in pain processing, have been reintroduced, but interestingly, the Special Issue was authored by another expert in the field [1] who utilized a different perspective to explore this issue. However, before turning our gaze to the insular function and its involvement in chronic pain, we annotated that this second edition can improve our information concerning neuropathic pain (NeuP) by dealing with the epigenetic changes occurring at the supraspinal level in different brain areas [2] and the maladaptive neuroplasticity and central mechanisms that underlie a neglected form of chronic pain that is identified as complex regional pain syndrome (CRPS) [3]. On the other hand, these heterogeneous contributions well reflect the “elusive” nature of pain and, in particular, NeP, which is characterized by multiple etiologies (e.g., traumatic, drug-therapy-associated, viral and endocrine causes), sensory alterations (e.g., paresthesia and numbness), the loss of function (e.g., motor function) and, above all, the partial efficacy or total inadequacy of the drugs used to treat this chronic condition [4]. 

The discussion of the “origin” of NeP often neglects the fact that Nep may have either central nervous system or peripheral causes. Likewise, the cognitive and affective aspects in terms of NeP should not be undervalued in explaining the progression from acute to chronic pain, and the paper by Rodrigues and colleagues [2] appropriately reminds us of the fact that specific brain areas are involved in both memory processing and affective features of pain, such as the hippocampus, prefrontal cortex, anterior cingulate cortex, ventral tegmental area and the nucleus accumbens. Thus, the persistence of pain memory is a factor responsible for the transition to chronic pain and the epigenetic changes that occur in different brain areas, a poorly investigated but substantial mechanism. These authors [2] investigated the mRNA expression of some key genes in different brain areas (e.g., hippocampus, medial prefrontal cortex, caudate-putamen and amygdala) and their association with the consolidation of NeuP. The key point concerns the epigenetic modifications, particularly DNA histone methylation, under the control of DNA methyltransferase (DNMTs) 1–3, so-called de novo DNMTs, and ten-eleven translocation dioxygenases (TET1–3) that have a role in passive and active demethylation [5]. The experimental induction of NeP produced differential TET1 (e.g., increased in the prefrontal cortex) or TET3 (e.g., reduced in the caudate-putamen and prefrontal cortex) patterns of expression as well as the downregulation of DNMT1 in selected brain areas, thus demonstrating the existence of differential gene expression throughout the neural circuits implicated in chronic pain. A study uncovered only a small piece of evidence concerning the still-unknown issue of DNA methylation in the establishment of chronic pain but revealed, in all their complexity, some epigenetic changes associated with NeP and different brain areas.

As mentioned before, there is well-accepted evidence concerning maladaptive neuroplasticity in terms of NeP pathophysiology, especially when the pain condition is evaluated at the central nervous system level. Nevertheless, much less is known about the role of the brainstem in pain processing, particularly in a prototypical painful condition, such as CRPS, whose precise etiology is still unknown [3,6]. The study conducted by Thoma and colleagues [3] used the nociceptive blink reflex (nBR) as a neurophysiological assessment of the nociceptive afferences (via the second-order brain stem neurons) and the response of the subject to repeated stimulation (i.e., habituation) as an index of sensitization. Thus, in this context, the study of habituation deficits may improve our ability to generate an early diagnosis and produce a better understanding of the involvement of the brainstem in the CRPS. As a matter of fact, and despite the small size of the sample of patients screened or the lack of control for medications, this work by Thoma and coworkers [3] is an interesting explorative study that provides evidence that an alteration related to the habituation to nBR can be used as a diagnostic marker of defective pain processing and CRPS. The first edition of the SI, “The Multiple Mechanisms Underlying Neuropathic Pain”, featured a study focused on the crosstalk between Toll-like receptor 5 and mu-opioid receptors (MORs) as functional interaction-mediating NeP-induced hyperalgesia [7]. Interestingly, the issue concerning opioid-mediated signaling as an anti-inflammatory strategy is discussed again through an experimental model of NeP (i.e., chronic constriction injury (CCI) in the work conducted by Vicario and colleagues [8]. In particular, the study explores the potential efficacy of opioid multitarget compounds, which are drugs targeting both MORs and delta-opioid receptors (DORs) that should allow reducing analgesic tolerance and MOR-inducing side effects. Within this context, the authors used the concomitant administration of a multitarget MOR-DOR agonist (i.e., LP2) and naloxonazine and naltrindole, as selective MOR and DOR antagonists, respectively, to evaluate the specific MOR and DOR analgesic contributions. However, the innovative point is the investigation of the mechanism of action involving gap junctions (GJs) and hemichannels (HCs) and their subunits connexins (Cxs) located on the plasma membranes as morphofunctional units for intercellular communication, especially in the mediation of astrocyte-to-microglia cell coupling and signaling. Moving from the idea that Cx43 is a connexin involved in inflammation and chronic pain after nerve injury, the study evaluated the hypothesis that the multitarget MOR-DOR agonist LP2 might achieve its analgesic efficacy by reducing Cx43 expression in the dorsal horns of the spinal cord and Cx43-mediated cell coupling. The results indicated that only concomitant MOR-DOR targeting can reduce Cx43-mediated heterocellular coupling between the astrocytes and microglia, thus providing new evidence about mechanisms of central sensitization and establishment of chronic neuropathy. A study also illustrated that the preclinical investigation of the analgesic potential provided by targeting the opioid system is not an exhaustive field of research in the management of chronic pain.

As discussed earlier, the first volume, and this second edition of the SI, deals with the investigation of the role of ICx in pain processing [1] but with a focus on neuroplasticity changes occurring during the maintenance of chronic pain and discusses the functional connectivity of ICx to other brain regions (e.g., anterior cingulate and prefrontal cortices, amygdala and the caudate-putamen) and the signaling systems (e.g., dopamine and oxytocin transmission) involved in different aspects of pain perception (e.g., sensory, emotional and cognitive). While the ICx is undeniably part of the brain network subserving, together with the somatosensory cortices I and II and the lateral thalamus, the sensory component of pain [9], studies relating to ICx function have also disclosed its higher activation in men rather than in women, thus highlighting marked sex differences in brain activation in response to painful stimulation and noxious stimuli [10]. An indication further corroborated by a recent study revealed significant gender-dependent disparities in the functional connectivity between different insular subregions [11]; therefore, some subdivisions of the ICx (e.g., dorsal dysgranular insula) were found to be greatly activated in male subjects. Furthermore, insular morphometry has been confirmed to be highly gender-dependent in addictive behaviors [12] and, considering the role of ICx in pain processing discussed in the present SI [1], there is evidence that the functional connectivity between different brain regions should be better investigated to improve our understanding of sex differences in pain processing. These considerations allow us to introduce the last manuscript published in this second volume, which concerns our investigation [13] related to the antinociceptive potential of the diabetic drug metformin (MTF) and its sex-dependent analgesic effects. Indeed, MTF efficacy can be considered quite different in male and female subjects, with a progressive loss of analgesia and a lack of durable antinociceptive effects in female animals, thus affecting its clinical use (or, at least, recommendations of use) for the management of NeP. The striking difference concerning the efficacy between the two sexes can be ascribed to multiple factors, among which, to very crucial morphological differences (e.g., neurofilaments, axon regeneration, autophagy of Schwann cells and early Wallerian degeneration). 

By collecting demonstrations that, depending on brain areas, DNA methylation is critical for the establishment of chronic pain [2], and that the brainstem is involved in pain processing (via the study of habituation to nBR) [3], that MOR-DOR multitargeting drugs can produce anti-inflammatory effects through astrocyte/microglia functional uncoupling [8], that the study of ICx can improve our knowledge not only of the different components of pain processing and experience [1] but also about the gender gap in pain susceptibility and response to therapy [13], we believe that this second volume of the SI “The Multiple Mechanisms Underlying Neuropathic Pain” has addressed many of the planned objectives.
